# The influence of emotion on keyboard typing: an experimental study using visual stimuli

**DOI:** 10.1186/1475-925X-13-81

**Published:** 2014-06-20

**Authors:** Po-Ming Lee, Wei-Hsuan Tsui, Tzu-Chien Hsiao

**Affiliations:** 1 Institute of Biomedical Engineering, National Chiao Tung University, Hsinchu, Taiwan; 2 Institute of Computer Science and Engineering, National Chiao Tung University, Hsinchu, Taiwan; 3 Department of Computer Science, National Chiao Tung University, Hsinchu, Taiwan; 4 Biomedical Electronics Translational Research Center and Biomimetic Systems Research Center, National Chiao Tung University, Hsinchu, Taiwan

**Keywords:** Emotion, Keyboard typing, Human subject experiment, International affective picture system

## Abstract

**Background:**

Emotion recognition technology plays the essential role of enhancement in Human-Computer Interaction (HCI). In recent years, a novel approach for emotion recognition has been reported, which is by keystroke dynamics. This approach can be considered to be rather desirable in HCI because the data used is rather non-intrusive and easy to obtain. However, there were only limited investigations about the phenomenon itself in previous studies. This study aims to examine the source of variance in keystroke typing patterns caused by emotions.

**Methods:**

A controlled experiment to collect subjects’ keystroke data in different emotional states induced by International Affective Picture System (IAPS) was conducted. Two-way Valence (3) × Arousal (3) ANOVAs were used to examine the collected dataset.

**Results:**

The results of the experiment indicate that the effect of emotion is significant (p < .001) in the keystroke duration, keystroke latency, and accuracy rate of the keyboard typing. However, the size of the emotional effect is small, compare to the individual variability.

**Conclusions:**

Our findings support the conclusion that the keystroke duration, keystroke latency, and also the accuracy rate of typing, are influenced by emotional states. Notably, the finding about the size of effect suggests that the accuracy rate of the emotion recognition could be further improved if personalized models are utilized. On the other hand, the finding also provides an explanation of why real-world applications which authenticate the identity of users by monitoring keystrokes may not be interfered by the emotional states of users. The experiment was conducted using standard instruments and hence is expected to be highly reproducible.

## Background

Graphics and the computing capabilities of computers have become powerful recently. However, a computer interactive application that does not understand or adapt to a users’ context, such as the emotion states of a user, could still lead to usability problems. Such an application could provide annoying feedback, interrupt users in an inappropriate situation, or increase the user’s frustration. Furthermore, it is also known that emotion can affect people with respect to their memory, assessment, judgment, expectations, opinions and even motor behaviors. Hence, it is crucial to consider the effect of emotions in modern usability studies: a computer could take advantage of the effect by presenting stimuli that sustain the desired emotions or, alternatively, avoid undesired emotions. Frustrated users could be guided to a different task, focus on a different aspect of the current task, or simply be advised to take a break.

In 1990s, Rosalind W. Picard, the mother of “Affective Computing”, began to propose and demonstrate her ideas about having computers identify a user’s emotion states and about possible improvements to the usability of computer applications [1]. Subsequently, many approaches for detecting users’ emotions have been demonstrated to be useful. For example, emotion recognition by facial expression, which aims to model visually distinguishable facial movements [2]; by speech, for which researchers utilize acoustic features such as pitch, intensity, duration, and spectral data [3]; and by physiological data, such as the heart rate and sweat [4].

Emotion recognition technology based on keystroke dynamics was not reported in the literature until Zimmermann P, Guttormsen S, Danuser B and Gomez P [5] first described this approach. These authors proposed an experiment designed to examine the effect of film-induced emotional states (PVHA, PVLA, NVHA, NVLA and nVnA (P = positive, N = negative, H = high, L = low, n = neutral, V = valence, A = arousal)) in subjects, with the keystroke dynamics in regard to keystroke rate per second, average duration of keystroke (from key-down until key-up event). However, they did not actually carry out the work described in their proposal. The use of keystroke dynamics for emotion recognition has two main advantages that make such the technique favorable. The two advantages are that it is non-intrusive and easy-to-obtain because the technique does not require any additional equipment or sensors other than a standard input device, which is the keyboard of a computer.

Later, numerous studies in the field of computer science have reported the development of emotion recognition technology based on keystroke dynamics. Vizer LM, Zhou L and Sears A [6] reported the use of ratios between specific keys and all keys to recognize task-induced cognitive and physical stresses from a neutral state. They achieved a classification rate of 62.5% for physical stress and 75% for cognitive stress. The key ratios could represent the frequencies of typing specific keys, which may increase or decrease due to the changes in emotional state. The analysis result was produced based on sophisticated Machine-Learning (ML) algorithms, and hence, the relationship between emotion and these ratios was not identified. Notably, most of the main streams of ML algorithms only produce models that are considered to be a black box, and do not produce readable^a^ models. The ML algorithms are usually used for building models from dataset that contains complex relationships which are not able to be identified by a traditional statistical model (e.g., t-test, ANOVA).

In 2011, Epp C, Lippold M and Mandryk RL [7] reported a result of building models to recognize experience-sampled emotional states based on keystroke durations and latencies that were extracted from a fixed typing sequence. The accuracy rates of classifying anger, boredom, confidence, distraction, excitement, focus, frustration, happiness, hesitance, nervousness, overwhelmed, relaxation, sadness, stress, and tired, with respect to two-class models^b^, were 75% on average. The study built models by using ML algorithms and a correlation-based feature subset attribute selection method [8]. Although the keystroke features that were used to build the model with the highest accuracy were provided, the relationship between emotion and keystroke dynamics, still, was not reported. Recently, more results related to classification on emotional data using similar feature set have been proposed. Alhothali A [9] reported the use of keystroke features that were extracted from arbitrarily typed keystroke sequences as reaching an 80% accuracy rate of classifying experience-sampled positive and negative emotional states. Bixler R and D’Mello S [10] demonstrated a 66.5% accuracy rate on average for two-class models in detecting boredom, engagement, and neutral states. The emotional data used were collected using the experience sampling method.

By applying ML methodology for building classification models from various datasets collected from different experimental setups, these studies have suggested that keystroke duration and keystroke latency can be used for model building. One therefore could hypothesize that the keystroke duration and latency may be different when subjects are in different emotional states. However, the details about the source of variance were never discussed in previous studies possibly due to the limitation of the adopted methodology. Hence, the current study aims to test the hypotheses that keystroke dynamics may be influenced by emotions.

The current study argues that the relationship between emotion and keystroke dynamics should not be too complex. By using a rigorous experiment setup, traditional statistical methods could be used to examine the variance and reveal the relationship, without the use of sophisticated ML algorithms. The study examines the variance of keystroke dynamics caused by emotions. Specifically, three hypotheses were tested. It is hypothesized that keystroke duration, keystroke latency, and the accuracy rate of a keyboard typing task are influenced by emotions. The study aims to answer two research questions. First, do the variance in the keystroke features that are ordinarily used for model building (i.e. keystroke duration, keystroke latency, accuracy rate) in previous studies exceeds significance level under different emotional states. Second, how large is the variance contributed by emotions in these keystroke features.

## Methods

### Ethics statement

This study is under the research project “A study of interactions between cognition, emotion and physiology (Protocol No: 100-014-E),” which was approved by the Institution Review Board (IRB) of the National Taiwan University Hospital Hsinchu Branch. Written Informed consents were obtained from all subjects before the experiment.

### Subjects

Twenty-seven subjects ranging in age between 19 and 27 (M = 21.5, SD = 2.3) performed keyboard typing tasks right after presented with emotional stimuli. The subjects were college students selected from a university in Taiwan, with normal or corrected-to-normal vision and normal range of finger movement. All the subjects self-reported that they were non-smoker, healthy, with no history of brain injury and cardiovascular problems.

### Experimental procedure

**Figure 1:**
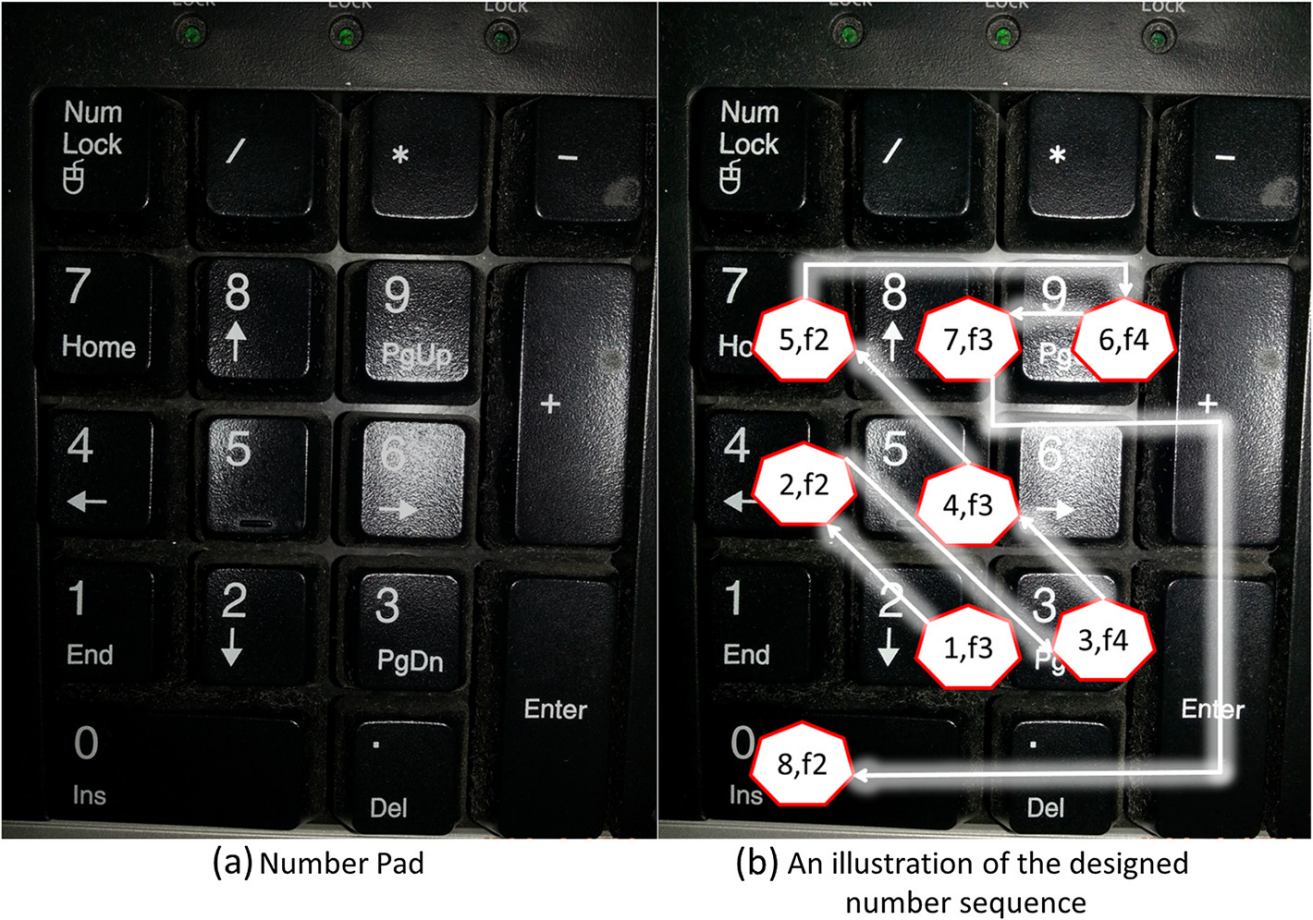
**An illustration of the designed target typing sequence.** The number pad in the keyboard used in our experiment, with an illustration of the design concept of our designed target typing sequence. The arrow shows the order of changes of the typing target. For those (x, y) pairs in the heptagons, x represents the order of a typing target and y represents the desirable finger (i.e. thumb (f1), index finger (f2), middle finger (f3), ring finger (f4), and little finger (f5) or pinky) that was used for typing the corresponding typing target.

A subject was instructed to type-in a target typing text “24357980” once immediately after the subject was presented with each of the International Affective Picture System (IAPS) [11] picture, for 60 trials. The experiment was conducted based on a simple dimensional view of emotion, which assumes that emotion can be defined by a coincidence of values on two different strategic dimensions that are, valence and arousal. To assess these two dimensions of the affective space, the Self-Assessment Manikin (SAM), an affective rating system devised by Lang PJ [12] was used to acquire the affective ratings.Each trial began with an instruction (“Please type-in the target typing text after the presentation of the next picture”) that was presented for 5 s. Then, the visual stimulus was presented for 6 s. After the presentation, the SAM with a rating instruction (“Please rate your feeling on both the two dimensions after typing the target typing text ‘24357980’”) was presented. The subject first typed-in the target typing text once, and then made their ratings of valence and arousal. A standard 15 s rating period was used, which allows ample time for subjects to make the SAM ratings. A computer program controlled the presentation and timing of the instructions and also the presentation of pictures. The keystroke data was recorded during the typing task. In addition to the 60 trials, 3 practice trials and a training section were applied prior to the experiment. Three pictures (a man, a snake, and newspapers) provided subjects with a rough range of the types of contents that were presented. After these practice trials was the training section, in which the subjects continually typed-in the target typing text (presented on the screen by blue text and gray background) using the number pad (shown in Figure 1(a)) that is located on the right side of a standard keyboard, for 40 s.

A number sequence was used as the target typing text instead of an alphabet sequence or symbols to avoid possible interference that can be caused by linguistic context on the subject’s emotional states. In all the various number sequences used in our pilot experiments [13], we found the existence of the difference in keystroke typing between the subjects in different emotional states. However, we also found that the relationship between the keystroke typing and emotional states may be different due to different keys that are typed and also the order of typing. A comparison of keystroke typing between emotional states using different number sequences may reduce the power of statistical tests (given a same number of trials). Hence, to conduct a more conservative comparison across emotion and to enhance the generalizability of this study, we decided to use a single number sequence that is designed to be general. In the current study, we designed the target typing text “24357980” to 1) be easy to type without requiring the subjects to perform abrupt changes in their posture, 2) have the number of digits fairly distributed on a number pad, and 3) encourage all of the subjects to maintain the same posture (i.e., in terms of finger usage) when typing the given sequence [13] (see Figure 1(b) for more detail). The time length of the experiment was designed to be as short as possible to avoid the subjects from being tired of typing on the keyboard. Note that all the subjects indeed reported that they were not fatigued after the experiment.

### Stimuli and self-report

The stimuli we used were 60 pictures selected from the IAPS database, which is being developed and distributed by the NIMH Center for Emotion and Attention (CSEA) at the University of Florida [11]. The IAPS is developed to provide a set of normative emotional stimuli for experimental investigations of emotion and attention and can be easily obtained through e-mail application. The IAPS database contains various affective pictures proved to be capable of inducing diverse emotions in the affective space [14]. The pictures we used as the stimuli were selected from IAPS database complying the IAPS picture set selection protocol described in [11]. The protocol includes the constraint about the number of pictures used in a single experiment, and the distribution of the emotions that are expected to be induced by the selected pictures. Stimulus order was randomized by a computer program for each subject, in order to balance the position of a particular stimulus within the series across the subjects.

The SAM is a non-verbal pictorial assessment that is designed to assess the emotional dimensions (i.e. valence and arousal) directly by means of two sets of graphical manikins. The SAM has been extensively tested in conjunction with the IAPS and has been used in diverse theoretical studies and applications [15-17]. The SAM takes a very short time to complete (5 to 10 seconds). For using the SAM, there is little chance of confusion with terms as in verbal assessments. The SAM was also reported to be capable of indexing cross-cultural results [18] and the results obtained using a Semantic Differential scale (the verbal scale provided in [19]). The SAM that we used was identical to the 9-point rating scale version of SAM that was used in [11], in which the SAM ranges from a smiling, happy figure to a frowning, unhappy figure when representing the affective valence dimension. On the other hand, for the arousal dimension, the SAM ranges from an excited, wide-eyed figure to a relaxed, sleepy figure. Ratings are scored such that 9 represents a high rating on each dimension (i.e. positive valence, high arousal), and 1 represents a low rating on each dimension (i.e. negative valence, low arousal).

### Apparatus

During the experiment, a subject sat on an office chair (0.50 × 0.51 m, height 0.43 m), in a small, quiet office (7.6 × 3.2 m) without people. The office was with window and the ventilation was guaranteed. The computer system (acer Veriton M2610, processor: Intel Core i3-2120 3.3G/3M/65 W, memory: 4 GB DDR3-1066, operating system: Microsoft Windows 7 Professional 64bit) used by the subject was put under a desk (0.70 × 1.26 m, height 0.73 m). The subject was seated approximately 0.66 m from the computer screen (ViewSonic VE700, 17 inch, 1280 × 1024 in resolution). The keyboard used by the subject was an acer KU-0355 (18.2 × 45.6 cm, normal keyboard with the United States layout, typically used for Windows operating system) connected to the computer system used through USB 2.0 communication interface. The distance between the center of adjacent keys (size: 1.2 × 1.2 cm) of the number pad used was 2 cm. Keyboard lifts (the two small supports at the back of the keyboard) which will raise the back of the keyboard for 0.8 cm when used, were not used in this experiment. The subject was sat approximately 0.52 m from the center of the number pad (i.e. the digit “5” of the number pad). The software designed for keystroke collection was developed using C# project built by using Visual Studio 2008 and was executed on the .NET framework (version 3.5) platform. The reason of using C# in developing this software is that Microsoft Windows operating systems provide more sufficient Application Programming Interfaces (APIs) for C# to detect keystroke-interrupt than for other programming language such as R, Matlab, Java, and Python.

### Data analysis

In total, 60 (trials) × 27 (subjects) = 1,620 rows of the raw data were collected during the experiment. To examine the keyboard typing patterns, single keystroke analysis [20] was applied to our raw data. Keystroke durations and keystroke latencies are ordinarily used in previous studies for single keystroke analysis [5,7,13]. The keystroke duration is the time that elapses from the key press to the key release, whereas the keystroke latency is the time that elapses from one key release to the next key press [21].

In our analysis, a sequence typed is a “correctly typed sequence” if the target typing text was correctly typed and “incorrectly typed sequence” if incorrectly typed. For example, if a subject typed “244357980”, in which the “4” at the 2^nd^ digit is misplaced, such that the sequence typed is considered as an incorrectly typed sequence. A pre-processing routine was applied to the raw data to separate all the correctly typed sequences from incorrectly typed sequences.

Keystroke duration and keystroke latency features were only extracted from the correctly typed sequences (90.2% of the 1620 samples). The extracted keystroke duration and keystroke latency features were submitted to two two-way 3 (Valence: negative, neutral, and positive) × 3 (Arousal: low, medium, and high) Repeat Measures ANOVAs [22], respectively. To analyse the accuracy rate of keyboard typing, the accuracy data (0 for incorrectly typed sequence and 1 for correctly typed sequence) of all the typed sequences was submitted to a two-way 3 (Valence: negative, neutral, and positive) × 3 (Arousal: low, medium, and high) Repeat Measures ANOVA.

The 9-point scale SAM ratings of the valence and arousal were translated into three levels of the ANOVA factor Valence and Arousal. The significance level α of the entire statistical hypothesis tests used in this paper was set to 0.05.

## Results

### Emotion inducement

**Figure 2:**
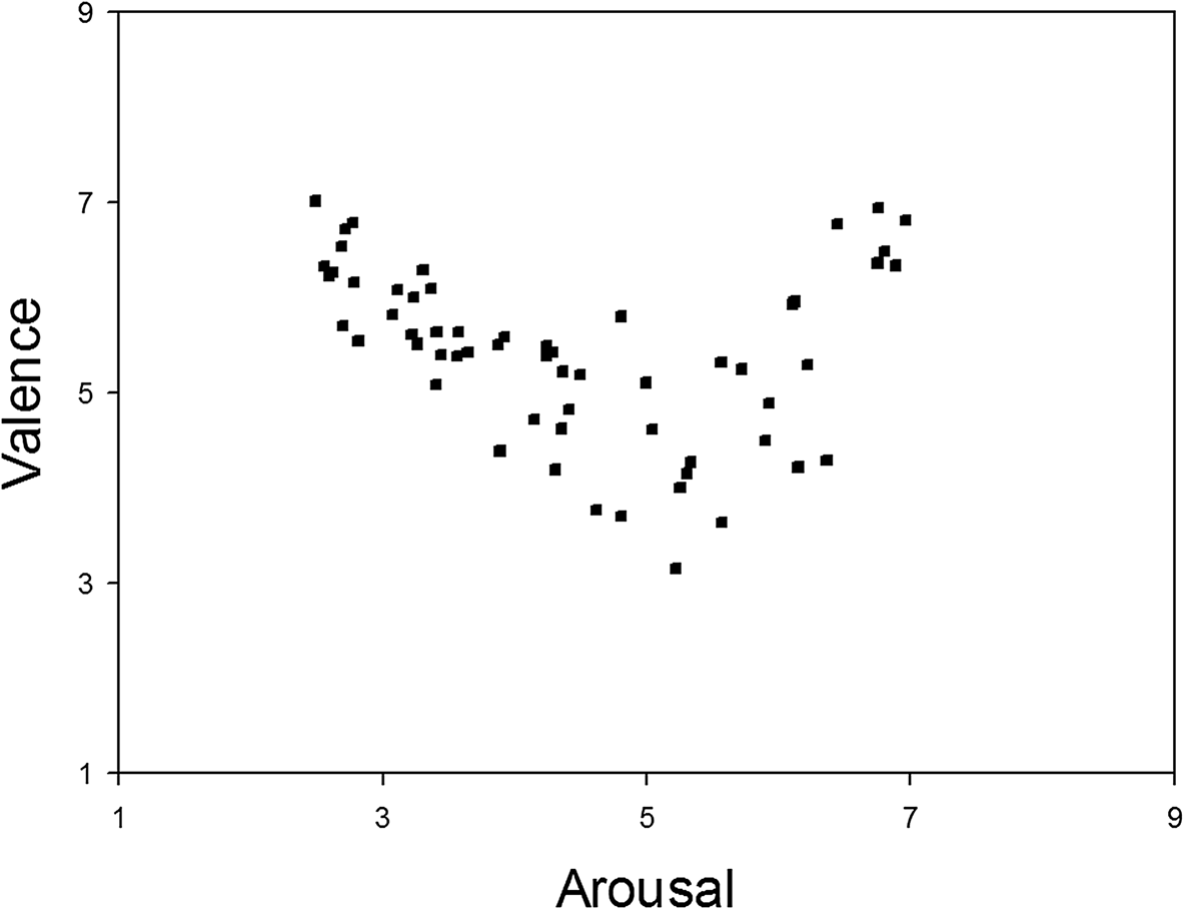
**Distribution of mean valence and arousal ratings elicited by the IAPS.** The distribution of the mean valence and arousal ratings elicited by IAPS pictures during the experiment.

The subjects have rated their feelings using the SAM on the dimensions of valence and arousal (see Figure 2). In Figure 2, each of the IAPS picture was plotted in terms of its mean valence and arousal rating. It is clear that the utilized pictures evoked reactions across a wide range of each dimension. The U-shaped relation between the valence and arousal indicates that these IAPS pictures elicited the subjects’ feelings of being annoyed or alarmed (i.e. reporting negative valence with medium arousal), but not being angry (i.e. reporting negative valence with high arousal) and not being tired, sad, or bored (i.e. reporting negative valence with low arousal). The mapping of the valence-arousal space to possible discrete emotional states was derived from previous studies [23,24].

### Influence of emotion on keystroke duration

The descriptive statistics of the influence of emotion on keystroke duration are provided in Table 1. This keystroke duration data was submitted to a two-way Repeat Measures ANOVA. The ANOVA results are provided in Table 2. Statistically significant difference was found in the main effect Valence. Valence by Arousal interaction was also significant. These results support the hypothesis that keystroke duration is influenced by valence. The percentage of the variability in the keystroke duration associated with the Valence (η^2^) is 64.28 (After removing the effects of individual differences).

**Table 1:** Descriptive statistics of keystroke duration under independent variables valence × arousal

**Valence**	**Arousal**	**Mean**	**Std. error**	**95% confidence interval**	
				**Lower bound**	**Upper bound**
Negative	Low	0.1049	0.0010	0.1028	0.1070
	Medium	0.0922	0.0010	0.0902	0.0943
	High	0.0946	0.0005	0.0936	0.0955
Neutral	Low	0.0944	0.0009	0.0926	0.0963
	Medium	0.0933	0.0012	0.0910	0.0956
	High	0.0942	0.0016	0.0910	0.0974
Positive	Low	0.0953	0.0013	0.0927	0.0979
	Medium	0.0950	0.0014	0.0922	0.0977
	High	0.0931	0.0007	0.0918	0.0945

**Table 2:** Repeated measures two-way ANOVA table for keystroke duration

**Source of variance**	**SS**	**df**	**MS**	**F**	**P**
Subjects	5.261	26	0.202		
Valence***	0.023	2	0.011	46.798	<.001
Error (Valence)	0.013	52	0		
Arousal	0.068	2	0.034	-54.353	1.00
Error (Arousal)	-0.033	52	-0.001		
Valence × Arousal***	0.062	4	0.015	26.161	<.001
Error (Valence × Arousal)	6.918	104	0.001		
Total	12.311	242			

Result of the 3 (Valence: negative, neutral, positive) × 3 (Arousal: low, medium, high) ANOVA. ***p < .001.

### Influence of emotion on keystroke latency

The descriptive statistics of the influence of emotion on keystroke latency are provided in Table 3. This keystroke latency data was submitted to a two-way Repeat Measures ANOVA. The ANOVA results are provided in Table 4. Statistically significant difference was found in the main effect Valence and Arousal. Valence by Arousal interaction was also significant. These results support the hypothesis that keystroke latency is influenced by emotional states. Specifically, keystroke latency can be influenced by both valence and arousal. The percentage of the variability in the keystroke latency associated with the Valence and Arousal (η^2^) are 23.75 and 52.77, respectively (After removing the effects of individual differences).

**Table 3:** Descriptive statistics of keystroke latency under independent variables valence × arousal

**Valence**	**Arousal**	**Mean**	**Std. error**	**95% confidence interval**	
				**Lower bound**	**Upper bound**
Negative	Low	0.1182	0.0030	0.1122	0.1243
	Medium	0.1472	0.0038	0.1396	0.1548
	High	0.1444	0.0022	0.1401	0.1488
Neutral	Low	0.1424	0.0035	0.1353	0.1495
	Medium	0.1544	0.0042	0.1461	0.1627
	High	0.1295	0.0051	0.1192	0.1397
Positive	Low	0.1347	0.0043	0.1261	0.1434
	Medium	0.1404	0.0045	0.1315	0.1493
	High	0.1316	0.0027	0.1262	0.1369

**Table 4:** Repeated measures two-way ANOVA table for keystroke latency

**Source of variance**	**SS**	**df**	**MS**	**F**	**P**
Subjects	26.452	26	1.017		
Valence***	0.112	2	0.056	8.096	0.0009
Error (Valence)	0.359	52	0.007		
Arousal***	0.351	2	0.176	29.052	<.001
Error (Arousal)	0.314	52	0.006		
Valence × Arousal***	0.524	4	0.131	12.772	<.001
Error (Valence × Arousal)	105.26	104	0.01		
Total	133.372	242			

Result of the 3 (Valence: negative, neutral, positive) × 3 (Arousal: low, medium, high) ANOVA. ***p < .001.

### Influence of emotion on accuracy rate

The descriptive statistics of the influence of emotion on accuracy data (0 for incorrectly typed sequence and 1 for correctly typed sequence) of all sequences typed are provided in Table 5. This accuracy rate data was submitted to a two-way Repeat Measures ANOVA. The ANOVA results are provided in Table 6. Statistically significant difference was found in the main effect Arousal. Valence by Arousal interaction was also significant. These results support the hypothesis that the accuracy rate of keyboard typing is influenced by arousal. The percentage of the variability in the accuracy rate associated with the Arousal (η^2^) is 20.42 (After removing the effects of individual differences).

**Table 5:** Descriptive statistics of accuracy rate under independent variables valence × arousal

**Valence**	**Arousal**	**Mean**	**Std. error**	**95% confidence interval**	
					**Lower bound**	**Upper bound**
Negative	Low	0.914	0.005	0.903	0.924
	Medium	0.784	0.008	0.767	0.801
	High	0.907	0.003	0.901	0.914
Neutral	Low	0.942	0.005	0.933	0.951
	Medium	0.879	0.008	0.863	0.895
	High	0.820	0.013	0.795	0.846
Positive	Low	0.862	0.009	0.844	0.881
	Medium	0.869	0.009	0.850	0.887
	High	0.855	0.005	0.844	0.866

**Table 6:** Repeated measures two-way ANOVA table for accuracy rate

**Source of variance**	**SS**	**df**	**MS**	**F**	**P**
Subjects	632.905	26	24.343		
Valence	5.58	2	2.79	1.352	0.2676
Error (Valence)	107.264	52	2.063		
Arousal***	19.067	2	9.533	6.67	0.0026
Error (Arousal)	74.324	52	1.429		
Valence × Arousal***	22.19	4	5.547	78.951	<.001
Error (Valence × Arousal)	1737.492	104	0.07		
Total	2598.821	242			

Result of the 3 (Valence: negative, neutral, positive) × 3 (Arousal: low, medium, high) ANOVA. ***p < .001.

## Discussion

Prior studies have highlighted the possibility of using keyboard typing data to detect emotions. Specifically, keystroke duration, keystroke latency, and accuracy rate of keyboard typing were used as input features for model building. These results have led to three hypothesized relationships. The relationship between keystroke duration and emotions, the relationship between keystroke latency and emotions, and the relationship between accuracy rate of keyboard typing and emotions. Hence, the current study tests these three hypothesized relationships. The results of our experiment using the fix target typing text and the 60 stimuli selected from the IAPS database support the hypothesis that the keystroke duration, keystroke latency, and also the accuracy rate of typing, are influenced by emotional states. The results further indicate that the keystroke duration is more sensitive to Valence, whereas the accuracy rate is more sensitive to Arousal. Moreover, the keystroke latency is affected by both Valence and Arousal, with these two variables interacts with each other.

It is worth to note that the size of the emotional effects that were found is small (see Tables 2, 4, and 6), compare to the individual variability. The finding suggests that although previous studies have built intelligent systems that act user-independently in detecting emotional states of users based on the keystroke dynamics, the accuracy rate of the detection could be further improved if personalized models are utilized. In addition, this finding also provides an explanation to that why real-world applications which authenticate the identity of users by monitoring keystrokes may not be interfered by the emotional states of users.

## Conclusions

The research questions about the three hypothesized relationships between emotions and keystroke dynamics are answered by using traditional statistical methods instead of sophisticated ML algorithms. The source of variance was examined and the emotional factors (in terms of valence and arousal) that affect keystroke duration, keystroke latency, and the accuracy rate of keyboard typing, were identified. To summarize, the evidence that were found supports all the three hypotheses.

## Endnotes

^a^A model that is readable means that the model is described clearly, with the relationship between independent variable and dependent variable identified and could be easily interpreted.

^b^A two-class model is the type of classification model that classifies instances into two classes (i.e. is an instance with the target label or not with the target label).

## Competing interests

The authors declare that they have no competing interests.

## Authors’ contributions

PM and TC conceived and designed the experiments and wrote the main manuscript text. WH prepared the data collection software and performed the experiments. All authors read and approved the final manuscript.
